# Non-apoptotic Regulated Cell Death Mechanisms in Sepsis and their Therapeutic Potential

**DOI:** 10.1007/s10753-025-02356-8

**Published:** 2026-02-02

**Authors:** Andrei Otto Mitre, Maria-Adriana Neag, Ioana Baldea, Gabriela Adriana Filip, Bianca Mitre, Alina Elena Parvu

**Affiliations:** 1 Pathophysiology, Department of Morpho-Functional Sciences, Faculty of Medicine, University of Medicine and Pharmacy “Iuliu Hațieganu”, Cluj-Napoca, 400012 Romania; 2 Pharmacology, Toxicology and Clinical Pharmacology, Department of Morpho-Functional Sciences, Faculty of Medicine, “Iuliu Hațieganu” University of Medicine and Pharmacy, Cluj-Napoca, 400012 Romania; 3 Physiology, Department of Morpho-Functional Sciences, Faculty of Medicine, IuliuHaţieganu University of Medicine and Pharmacy, Clinicilor 1, Cluj-Napoca, 400006 Romania; 4 Immunology and Allergology Department, “Octavian Fodor” Institute of Gastroenterology and Hepatology, Cluj-Napoca, 400162 Romania

**Keywords:** Sepsis, Cell death, Pyroptosis, Necroptosis, Ferroptosis

## Abstract

Regulated cell death (RCD) plays an important role in the pathophysiology of sepsis. Traditionally dominated by the concept of apoptosis, the understanding of cell death has significantly expanded to include a range of highly regulated, non-apoptotic mechanisms such as ferroptosis, necroptosis, pyroptosis, parthanatos, and NETosis. This review describes recent advances in the molecular pathways underpinning these RCDs and explores their contributions to inflammatory tissue damage during sepsis and ischemia/reperfusion injury. We highlight the key molecular drivers and signalling cascades for each form of RCD and how thei could interact during sepsis. Also, we present the effects of commonly used medication during sepsis and septic shock in the intensive care unit on RCD. By integrating current evidence, we propose that therapeutic modulation of RCD offers a promising avenue for reducing organ dysfunction and mortality in critical illness or could pose as biomarkers in evaluating the sepsis-associated organ injury.

**Graphical Abstract:**

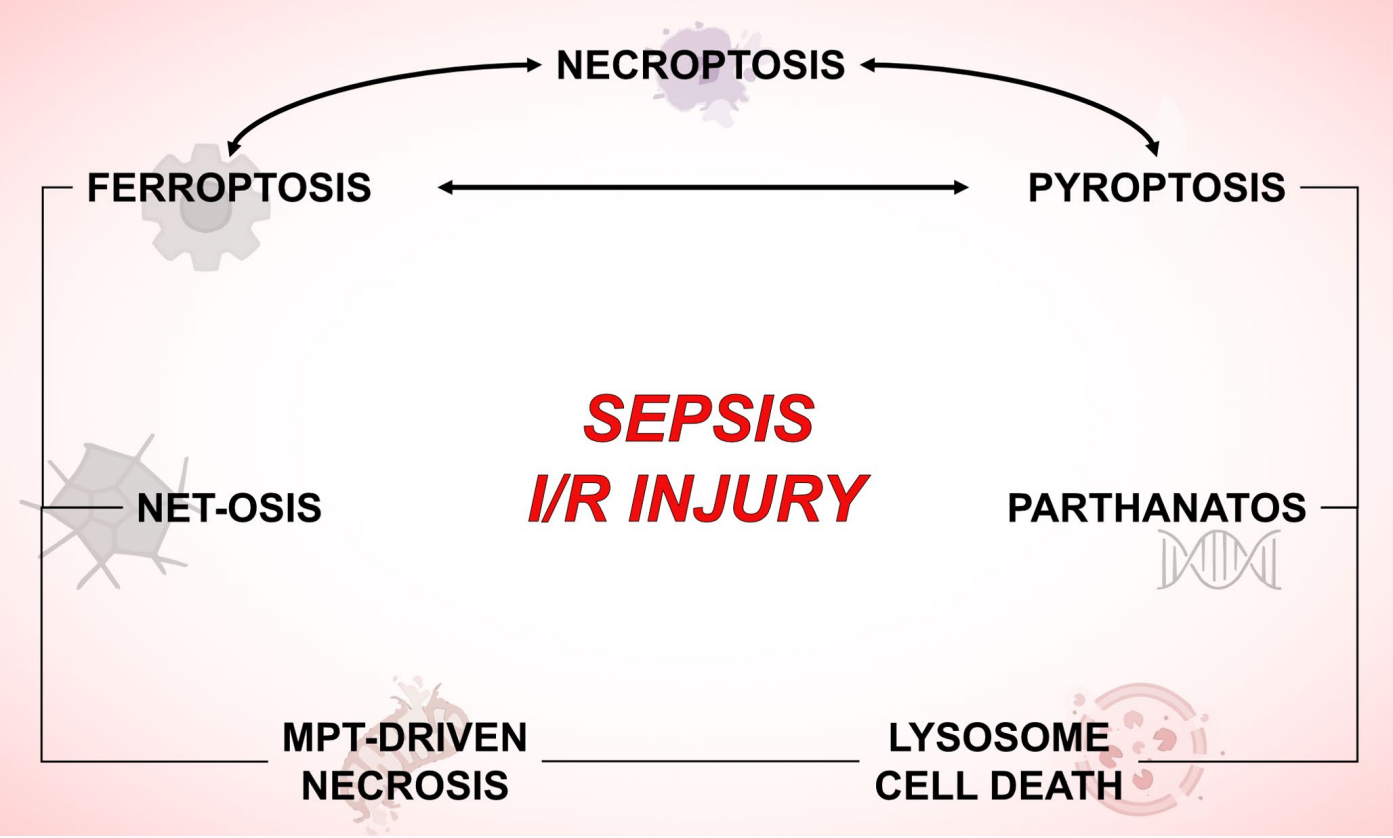

## Introduction

Sepsis is a life-threatening condition characterized by a dysregulated host response to infection, that can cause systemic inflammation, tissue damage and potential organ failure. It remains a major cause of morbidity and mortality worldwide, particularly in critically ill patients. The condition is triggered by infections that activate the innate immune system, leading to excessive production of inflammatory mediators, such as cytokines and reactive oxygen species (ROS), and endothelial dysfunction. This hyperactive response contributes to vascular leakage, impaired oxygen delivery and metabolic derangements, all of which play a role in the progression of organ dysfunction [1, 2].

Oxidative stress plays a central role in the pathophysiology of sepsis. Under normal conditions, ROS act as signalling molecules that help regulate immune responses and pathogen clearance. However, in sepsis, an excessive production of ROS overwhelms the body’s antioxidant defences, leading to oxidative damage of lipids, proteins and DNA. Mitochondrial dysfunction exacerbates this oxidative imbalance, resulting in cellular energy failure and further contributing to organ dysfunction. The inflammatory response in sepsis also amplifies ROS production, creating a vicious cycle that drives disease progression [3]. Targeting oxidative stress and inflammation through antioxidant therapies and immune modulation is an area of ongoing research aimed to improve sepsis outcomes [4].

Regulated cell death (RCD) is a controlled process by which cells undergo programmed elimination in response to specific signals. In sepsis, dysregulated RCD contributes to immune dysfunction and organ failure. Unlike accidental cell death, which occurs due to physical or chemical injury, RCD is mediated by distinct molecular pathways that help maintain tissue homeostasis and remove damaged or infected cells [5]. Several non-apoptotic forms of RCD are associated with increased inflammatory response including necroptosis, pyroptosis, ferroptosis, parthanatos, MPT-driven necrosis, NETosis and lysosome dependent cell death. Targeting RCD pathways with specific modulators (Table 1) has emerged as a potential therapeutic strategy to mitigate sepsis-induced damage and improve patient outcomes [6].

**Table 1:** Non-apoptotic cell death inhibitors. Abbreviations: ACSL4, acyl-CoA synthetase long-chain family member 4; GPX4, glutathione peroxidase 4; MLKL, mixed lineage kinase domain Like; NLRP, Nucleotide-binding oligomerization domain, leucine rich repeat and Pyrin domain containing; PARP1, poly(ADP-ribose) polymerase; RIPK, Receptor-Interacting protein kinase

Cell death mechanism	Inhibitor	Mechanism of inhibition	Reference
Necroptosis			
	RIPK1 inhibitor	Necrostatin-1 (Nec-1) and Nec-1s	[7]
	RIPK1 inhibitor	GSK2982772 (GSK’772)	[8]
	RIPK3 inhibitor	GSK’843; GSK’872;	[9–11]
	MLKL inhibitor	Necrosulfonamide; TC13172; GW806742X	[12–14]
Pyroptosis			
	Caspase-1 inhibitor	VX-765; Belnacasan (VX-740);	[15, 16]
	Gasdermin D inhibitor	Disulfiram; LDC7559	[17, 18]
	NLRP3 inflammasome inhibitor	MCC950; CY-09; Oridonin	[19–21]
Ferroptosis			
	Lipid peroxidation inhibitor	Ferrostatin-1; Liproxstatin-1;	[22, 23]
	GPX4 inhibitor	RSL3	[24]
	Iron chelator	Deferoxamine	[25]
	ACSL4 inhibitor	Rosiglitazone	[26]
Parthanatos			
	PARP1 inhibitor	PJ34; Olaparib; Rucaparib; Talazoparib	[27–30]

This review will explore the major types of non-apoptotic RCD pathways linked to inflammatory response. It will also examine the role of RCD in sepsis, the impact of inhibiting these pathways, and how commonly used medications in intensive care units (ICUs) influence these forms of cell death and in the end we will briefly discuss the current clinical implications of this research area.

## Non-apoptotic Inflammatory Cell Death Mechanisms and Sepsis

### Necroptosis

**Fig. 1:**
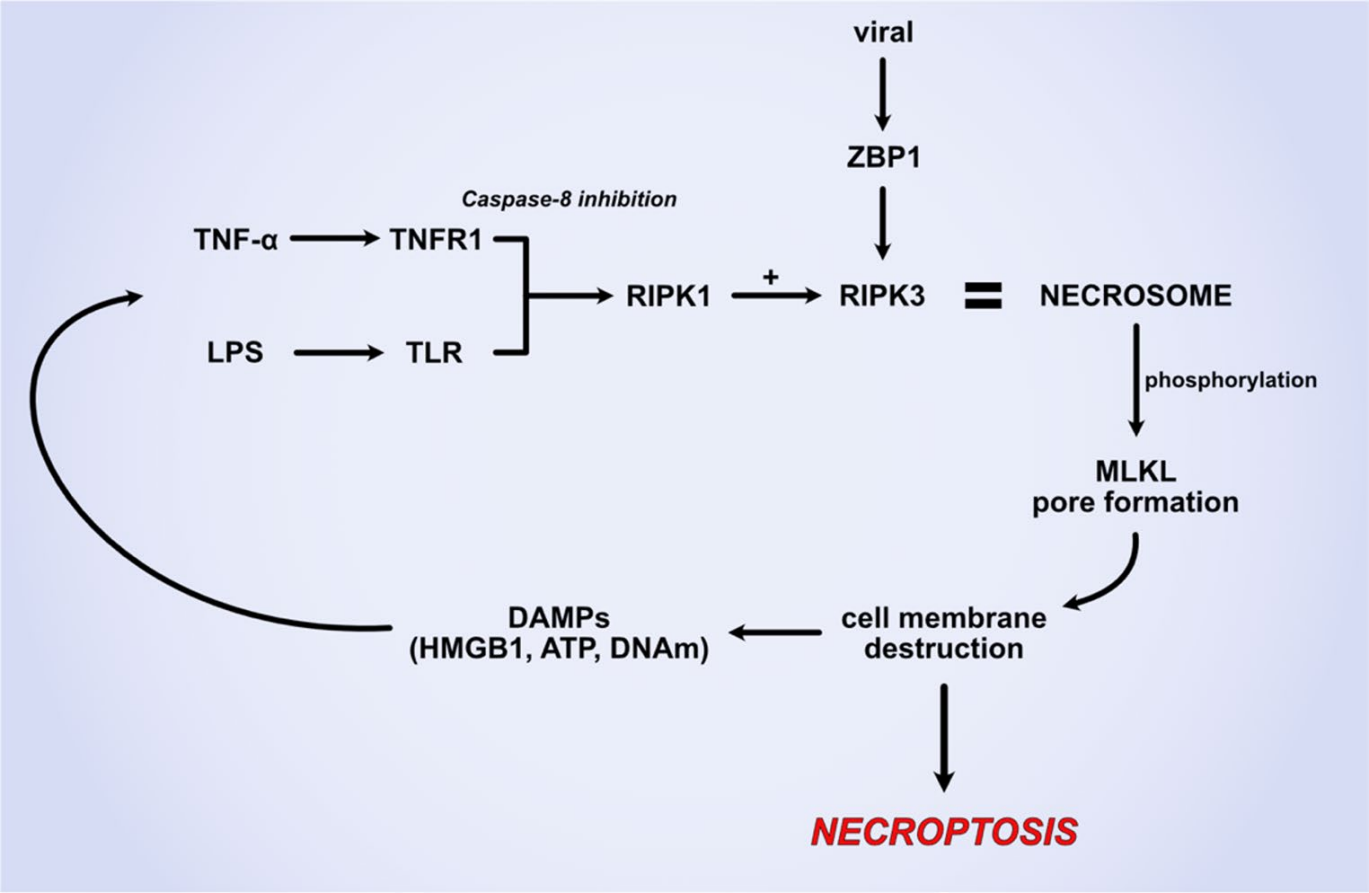
Necroptosis pathway. Inflammatory mediator TNF-α and LPS trigger in cells with caspase-8 inhibited pathways the formation of the RIPK1 + RIPK3 necrosome, that leads to the phosphorylation of MLKL molecules and the formation of the MLKL pore. This leads to cell membrane destruction and necroptosis that is further amplified by the intracellular release of DAMPs. *Abbreviations*: ATP, adenosine triphosphate; DAMPs, damage-associated molecular patterns; HMGB1, high-mobility group box 1; LPS, lipopolysaccharide; MLKL, mixed lineage kinase domain-like protein; RIPK, receptor-interacting protein kinase; TLR, Toll-like receptor; TNF-α, tumour necrosis factor alpha; TNFR1, tumour necrosis factor receptor 1; ZBP, Z-DNA binding protein

Necroptosis is a programmed form of necrotic cell death that is essential in defence mechanisms against viral infections. It is also involved in neurodegenerative diseases, ischemia-reperfusion injuries, cancer and inflammatory conditions including sepsis (Fig. 1) [31–33]. It is activated when the apoptosis mediator caspase-8 is inhibited, shifting the normal apoptotic-cascade towards necroptosis [6]. Necroptosis is initiated by the activation of tumour necrosis factor (TNF) receptor 1, toll-like receptors or other death receptors including nucleic acid sensors, retinoic acid receptor responder 3 and adhesion receptors [34]. Under caspase-8 inhibition and via different pathways depending on stimuli, receptor interacting protein kinase 1 (RIPK1) is activated and interacts with RIPK3 forming the necrosome complex. The necrosome facilitates mixed lineage kinase domain like pseudokinase (MLKL) phosphorilation that undergoes oligomerization and forms pores in the plasma membrane, leading to ion influx, osmotic swelling and cell death [35]. These effects lead to the release of more damage-associated molecular patterns (DAMPs) that activate pattern recognition receptors and amplify the inflammatory response, as well as inflammasome activation and ROS production, causing further oxidative stress, inflammation and eventually cell death.

Because of its central role in inflammatory propagation, necroptosis is a key contributor to tissue injury, immune dysfunction and organ failure, but also to pathogen clearance [36]. A study of ICU sepsis patients’ blood levels of RIPK3 found that the necroptosis marker was associated with higher mortality in these patients [37]. Also, a bioinformatical analysis revealed that necroptosis-related differentially expressed genes are correlated with sepsis-induced myocardial dysfunction and could be used as future biomarkers [38].

Excessive cytokine production seen in sepsis, especially IFN-γ, IL-1β and TNF-α, are involved in necroptosis activation and propagation of the inflammatory response [39]. Further release of DAMPs accentuates the inflammatory response, but can also trigger other cell death pathways such as pyroptosis via NLRP3 inflammasome activation [40]. Pyroptosis can be activated by both RIPK3 and MLKL via a gasdermin D (GSDMD) mediated mechanism [41].

The central molecule of necroptosis is RIPK3. In a RIPK3 deficient mouse model of sepsis, TNF-α and IL-6 circulating levels were decreased when compared with non-deficient controls. Also, RIPK3 deficient mice with sepsis showed a reduced number of neutrophils and natural killer cells in the liver. This inhibition of necroptosis led to the combined effect of prolonged survival of septic rats and reduced organ injury [42]. Similar results were seen in a rat model of neonatal sepsis where RIPK1 inhibitor necrostatin-1 reduced serum cytokines levels and improved the histological aspect of the lung and increased survival [43]. Necrostatin-1 administered in a pig model of LPS-induced sepsis reduced necroptosis, liver inflammation and injury [44]. However, besides being involved in the induction and propagation of the inflammatory response, necroptosis could potentially reduce it as well. In a *Staphylococcus aureus* inflammation model, necroptosis played a key role in limiting the cytokine production triggered by infection [45]. Also, via a similar mechanism intracellular pathogens could escape from immune clearance [46].

Necroptosis inhibition is not only beneficial, as it can shift the balance of the inflammatory response towards other forms of cell death. In a cecal ligation and puncture (CLP) model of sepsis, necrostatin-1 inhibition of necroptosis led to an accelerated apoptosis pathway and increased liver injury and reduced survival of the septic animals [47].

Overall, necroptosis plays a multifaceted role in sepsis, acting both as a driver and inhibitor of inflammation. Identifying reliable biomarkers is crucial to determine which cell types are involved and the optimal timing during sepsis progression when targeting necroptosis could be beneficial for the patients.

### Ferroptosis

**Fig. 2:**
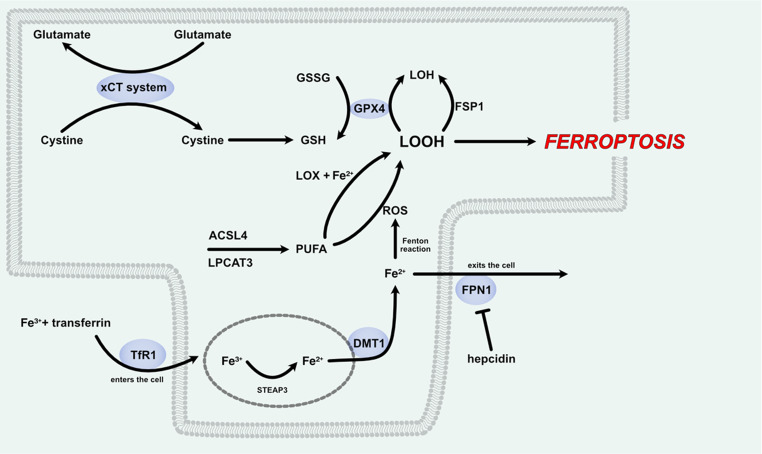
Ferroptosis pathway. Ferric iron enters the cell via TfR1, where the complex is reduced to ferrous iron that is then involved in the Fenton reaction, producing ROS. ROS oxidises PUFAs and lead to the formation of lipid peroxides. In the absence or depletion of antioxidant mechanisms, especially the xCT-Cystine-GSH-GPX4-mediated antioxidant system, lipid peroxides cause cell membrane destruction and ferroptosis. In sepsis this process is accentuated by the increased intracellular ferrous iron, as FPN1 is inhibited by increased hepcidin levels. *Abbreviations*: ACSL4, Acyl-CoA Synthetase Long-Chain Family Member 4; DMT1, Divalent Metal Transporter 1; Fe²⁺, Ferrous Iron; Fe³⁺, Ferric Iron; FPN1, Ferroportin 1); FSP1, Ferroptosis Suppressor Protein 1; GPX4, Glutathione Peroxidase 4; GSH, Reduced Glutathione; GSSG, Oxidized Glutathione; LOH, Lipid Alcohol; LOOH, Lipid Hydroperoxide; LOX, Lipoxygenase; LPCAT3, Lysophosphatidylcholine Acyltransferase 3; PUFA, Polyunsaturated Fatty Acid; ROS, Reactive Oxygen Species; STEAP3, Six-Transmembrane Epithelial Antigen of Prostate 3; TfR1, Transferrin Receptor 1; xCT, Cystine/Glutamate Antiporter Subunit SLC7A11

Ferroptosis was first described as an iron-dependent form of oxidative cell death [48]. Now ferroptosis can be described as a form of cell death that is triggered by lipid peroxidation in the presence of iron imbalance and failure of intracellular ROS scavenging systems [49]. There are several pathways that can lead to ferroptosis, such as cystine-GSH-GPX4 pathway, the poly-unsaturated fatty acids (PUFA) pathway and the iron-pathway (Fig. 2).

In the cystine-GSH-GPX4 pathway, L-cystine enters the cell by exchange with glutamate via the xCT exchanger system composed of Solute Carrier Family proteins, namely SLC7A11 and SLC3A2 carriers [50]. L-cystine is then transformed by γ-glutamyl cysteine synthase into γ-glutamyl cysteine and then into reduced glutathione (GSH) that acts as a ROS scavenger [51]. GSH can also be used by glutathione peroxidase 4 (GPX4), a member of the selenoprotein family, to reduce peroxides to alcohols [52]. This is one of the protective mechanisms against the formation of lipid peroxides (LOOH), the main feature of ferroptosis. GPX4 is required for the expansion phase of T cells and survival, thus playing an important role in cell-mediated immunity [53]. Nuclear factor erythroid 2-related factor 2 (Nrf2) is a transcription factor that regulates antioxidant responses, including the production of GPX4, the key enzyme involved in ferroptosis modulation [54, 55]. PUFAs are esterified by acyl-CoA synthetase long-chain fatty member 4 (ACSL4) and lysophosphatidylcholine acyltransferase 3 (LPCAT3) to polyunsaturated phospholipids (PUFA-PL), mainly phosphatidylethanolamine, that are then oxidized into LOOH by lipoxygenases and Fe^2+^ [56–58]. As mentioned before, this process can be limited by the GPX4 antioxidant system to prevent ferroptosis. In GPX4-knockout models another regulatory pathway was described. This involves the transformation of Ubiquinone/alpha-tocopheryl into Ubiquinol/alpha-tocopherol by the ferroptosis suppressor protein 1 (FSP1) [59]. Iron enters the cell freely via divalent metal transporter 1 (DMT1) or bonded with transferrin via endocytosis using transferrin receptor 1 (TfR1) as a receptor. Inside the endocytosis particle it is transformed by 6-transmembrane epithelial antigen of the prostate 3 (STEAP3) from Fe^3+^ into Fe^2+^ and then it enters the cell plasma via DMT1. Excess ferrous iron can leave the cell only via ferroportin (FPN) [60]. Fe^2+^ freely available in the plasma creates the labile or free iron pool that activates iron-regulatory proteins and bind to the iron response elements of mRNAs, inducing it’s stabilization or degradation and regulates metabolic processes including DNA synthesis, cell cycle progression and angiogenesis [61]. Part of the free iron binds to poly r(C)-binding protein 1 and 2 to form ferritin deposits and another part will bind to hydrogen peroxide in the Fenton reaction to form ROS that are then causing lipid peroxidation [62].

During sepsis, ferroptosis plays a dual role. It acts as a pathogen killing mechanism, but also as an anti-inflammatory mechanism, causing immune cells death, promoting bacterial invasion and tissue injury [63]. Because iron is an important micronutrient for bacterial division, the human body evolved to limit iron supply to the pathogens. In sepsis iron metabolism is altered [64], its availability towards bacterial use is reduced by sequestering iron into the cells. However, by raising its intracellular concentration it increases the risk of creating ROS and propagating ferroptosis [65]. During the inflammatory reaction cytokines trigger transferrin receptors expression favouring transferrin-iron complex internalization. The non-heme iron transporters like lactoferrin and lipocalin-2 are also elevated in sepsis, contributing to iron sequestration [66, 67]. In an animal model of doxorubicin toxicity, iron accumulation increased mitochondrial ROS production and inflammation [68, 69]. Other important enzymes involved in the sepsis pathophysiology that require iron as a cofactor are cyclooxygenases (COXs) and lipoxygenases (LOXs) [70]. They play a central role in the formation of proinflammatory mediators involved in sepsis-induced inflammatory response propagation [57, 71]. Iron levels could potentially promote these enzymes and therefore inflammation, but the exact mechanism is not yet elucidated [72]. However, the real importance of this mechanism is probably low, as clinical trials using COX inhibitors showed no significant improvement in sepsis survival [71].

FPN is essential to iron export from the cell and it is downregulated in sepsis, causing iron accumulation and increased ferroptosis [73]. Hepcidin regulates FPN levels by binding to FPN and causing its degradation [74]. Hepcidin levels are elevated in acute illnesses and in bacteraemia, and it has been proposed as a sepsis biomarker [75–77]. This mechanism is largely associated with the imbalance of inflammatory mediators, especially IL-6, IL-22 and IL-1β [78]. Its inhibition could be of potential benefit by allowing FPN export of iron outside the cells [79]. However, normal levels of FPN might not be beneficial against bacteriemia. A study on African population showed that a FPN variant that is more resistant to hepcidin degradation provided no beneficial effects in protecting against all-cause bacteriemia [80].

Nrf2 is an important transcription factor in sepsis and ferroptosis because of its role to regulate the production of GPX4. In septic models, Nrf2 supplementation showed improved organ outcomes, reduced ferroptosis and reduced oxidative stress, while Nrf2 knockout models with sepsis had increased organ damage and inflammation [81, 82]. Therefore, increasing Nrf2 expression could be a beneficial treatment strategy in sepsis [83, 84]. As mentioned before, under GPX4 deficient conditions, FSP1 activation by various pharmaceutical strategies can inhibit ferroptosis and organ damage by reducing oxidative stress making both these mechanisms important targets for future studies in order to reduce ROS and ferroptosis [85, 86].

### Pyroptosis

**Fig. 3:**
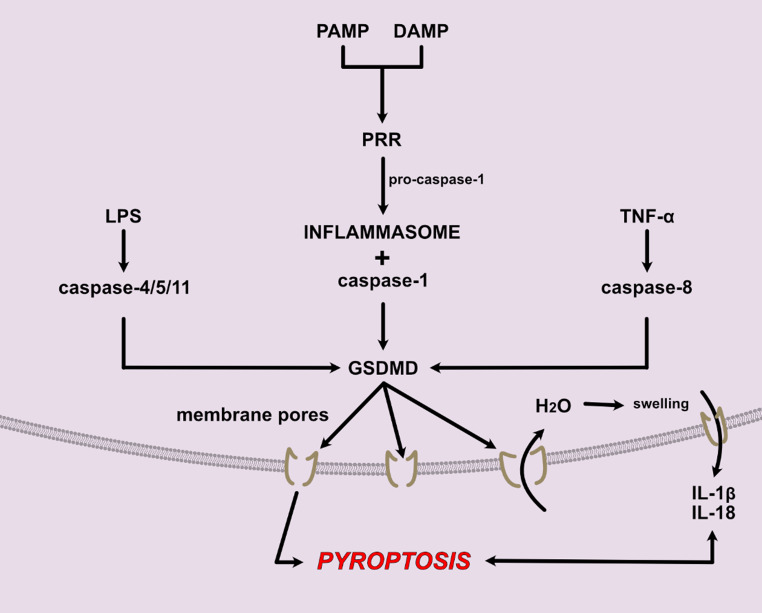
Pyroptosis pathway. Pyroptosis main trigger is the formation of GSDMD pores in the cell membrane, which leads to water entering the cell, swelling and ultimately cell lysis. This releases pro-inflammatory molecules IL-1β and IL-18 that further amplify the process. GSDMD pores can be trigger by a PAMP/DAMP-induced pathway, with the formation of the inflammasome or via LPS or TNF-α pathways. *Abbreviations*: DAMP, Damage-Associated Molecular Pattern; GSDMD, Gasdermin D; IL, Interleukin; LPS, Lipopolysaccharide; PAMP Pathogen-Associated Molecular Pattern; PRR, Pattern Recognition Receptor; TNF, Tumour Necrosis Factor

Pyroptosis is a form of regulated cell death driven by inflammatory responses, distinct from apoptosis and necroptosis [87]. Pyroptosis was first described as a caspase-1 mediated defence strategy during *Shigella flexneri* infections in macrophages and later the cell death name was used to describe the cell death of macrophages infected with *Salmonella typhimurium* [88–90]. By promoting the elimination of infected or damaged cells, pyroptosis contains the infection and alerts the immune system to the presence of danger. However, its unregulated activation can lead to excessive inflammation and tissue damage, contributing to the development of inflammatory and immune-mediated diseases [91, 92]. In recent years it has emerged as a key player in the pathogenesis of sepsis. By releasing DAMPs and pro-inflammatory cytokines, pyroptotic cells contribute to the cytokine storm that underpins sepsis progression, leading to widespread organ dysfunction (Fig. 3) [93].

The molecular mechanisms of pyroptosis revolve around the activation of caspases (caspase-1, caspase-4, caspase-5, and caspase-11) that cause the cleavage of gasdermin proteins which disrupt cellular membranes and trigger inflammatory responses. These pathways can be broadly classified into the canonical pathway, mediated by caspase-1, and the non-canonical pathway, mediated by caspase-4, −5, and − 11, as well as several other pathways that culminate in the formation of gasdermin pores and the release of pro-inflammatory cytokines such as IL-1β and IL-18 [94].

The canonical pyroptosis pathway is initiated by pathogen-associated molecular patterns (PAMPs) and DAMPs that activate inflammasomes, most notably NLRP3, AIM2, NLRC4, and Pyrin [95]. Upon activation, the inflammasome recruits procaspase-1 via the adapter protein apoptosis-associated speck-like (ASC). ASC has two interaction domains, a PYRIN domain (PYD), and a caspase-recruitment domain (CARD) that further auto-activates procaspase-1 through proximity-induced cleavage, generating active caspase-1 [96–98]. Caspase-1 activates pro-IL-1β and pro-IL-18 into their mature forms and activates GSDMD by cleavage of the N-terminal fragment that then binds to the phosphatidylinositol phosphates and cardiolipin from the cell membrane, forming pores through which H_2_O enters the cell, causing osmotic cell lysis [99, 100]. This releases intracellular content, including previously activated IL-1β and IL-18 that further amplify the inflammation.

The non-canonical or caspase-1-independent pathway is induced by caspases − 4 and − 5 (and caspase 11 in rodents) in response to lipopolysaccharide (LPS) released from Gram negative bacteria, independently from TLR4 activation [101–103]. These caspases directly trigger GSDMD activation and pyroptosis. Other mechanisms that cause GSDMD or gasdermin E (GSDME) activation and pore formation leading to pyroptosis have also been described, such as caspase-3 activation by chemotherapy drugs or TNF-α, caspase-8 activation by Yersinia, or TAK1 inhibition and gasdermin C (GSDMC) activation by hypoxia via PD-L1 [104–107].

Pyroptosis plays a critical dual role in the pathogenesis of sepsis. By releasing IL-1β, IL-18, and DAMPs it exacerbates systemic inflammation leading to organ damage. It can also serve as a defence mechanism by aiding in the elimination of pathogens; however, over time, it may contribute to immune paralysis [108, 109]. Persistent pyroptosis can deplete immune cells, weakening host defences and pyroptosis-induced release of DAMPs can lead to T-cell exhaustion and dysfunction, further compromising the immune system’s ability to combat new pathogens [110]. In a sepsis CLP mouse model, upregulation of peroxisome proliferator-activated receptor gamma (PPARγ) was found to decrease serum markers of liver injury and improve survival rates. This protective effect was attributed to the inhibition of hepatocyte pyroptosis via suppression of the ROS/TXNIP/NLRP3 signaling pathway [111]. Macrophage activation via HMGB1 and pyroptosis activation mediates part of the acute liver injuries seen in septic-livers [112]. Pyroptosis inhibition in liver macrophages reduced inflammation and the M1/M2 macrophage polarization therefore diminished the sepsis-associated liver injury [113].

In sepsis-induced lung injury, caspase-11 has been shown to participate in endothelial pyroptosis. Caspase-11 knockout mice exhibited reduced inflammation and lung injury after CLP, indicating that caspase-11 was involved in the pathogenesis of sepsis-induced lung injury [114]. Furthermore, inhibiting NLRP3 inflammasome activation in alveolar macrophages alleviated lung tissue injuries and reduced pro-inflammatory cytokine release in CLP-induced mice [115]. Renal-induced pyroptosis drives inflammatory pathways and is involved in sepsis-induced acute kidney injury [116]. Pyroptosis inhibition reduced the level of inflammatory cytokines in renal tissue samples and improved sepsis-outcomes in rodent models [117–120]. Endothelial cell pyroptosis contributes to vascular dysfunction in sepsis. Conditional knockout of caspase-11 in endothelial cells reduced endotoxin-induced pyroptosis, pulmonary edema and neutrophil accumulation [121]. Targeting signalling pathways like SP1/reticulocalbin-2 (RCN2)/ROS has also been shown to regulate endothelial pyroptosis, presenting a potential target for therapeutic intervention [122]. In a model of sepsis-induced myocardial depression (SIMD) the NLRP3 inflammasome and IL-1β levels are significantly elevated in cardiomyocytes. Studies have shown that NLRP3 knockout mice exhibit reduced cardiovascular damage and lower plasma levels of IL-1β and IL-6 compared to wild-type mice, indicating that NLRP3 is involved in SIMD [123]. Interventions targeting the TXNIP-NLRP3 interaction have been shown to decrease active caspase-1, IL-1β, and IL-18 levels, thereby alleviating cardiovascular inflammation in SIMD models [124].

Compare with caspse 11 in mice, caspase 4 is more sensitive to LPS and can trigger the secretion of IL-1β and IL-18 and the activation of caspase-1 [125]. Also, *B. pseudomallei* infection triggers the release of caspase 4 that will aid in the clearance of pathogens, but, as discussed before, can also lead to an altered inflammatory response and cause organ damage [126]. Similarly, caspse 5 and caspase 4 are essential for heme-induced IL-1β release [127].

In critically ill patients, caspase 5 gene expression remains elevated, while caspase 4 levels decrease during the disease. This associates the lower levels of caspase 4 with immunosuppression and organ failure. GSDMD levels were also declined, indicating that caspase 4 reduction reduced inflammasome activity and thus hindered the innate host response [128]. GSDMD activation is not only associated with pyroptosis, but can also trigger the formation of neutrophil extracellular traps (NETs) and lead to NETosis and sepsis-induced coagulopathy and worse outcomes for septic patients [129]. Its inhibition could prevent multiple organ dysfunction and potentially improve patients outcome [130].

### Parthanatos

Parthanatos is a poly (ADP-ribose) polymerase 1 (PARP-1) dependent programmed form of cell death that is distinct from apoptosis and necroptosis [131]. Under normal circumstances, mild DNA damage triggers PARP-1 activation which cleaves NAD + into nicotinamide and ADP-ribose that binds to acceptor proteins near the damage site. This forms poly (ADN-ribose) (PAR) and leads to DNA repair and cell survival [132]. However, when the DNA fragmentation is extensive, the excessively formed PARP-1 creates a large amount of PAR, leading to mitochondrial dysfunction. PARP-1 catalyses the synthesis of PAR chains using NAD + as substrate, which leads to NAD + depletion and ATP consumption, causing an energy crisis within the cell. The formed PAR-polymers translocate to the mitochondria, where they induce mitochondrial outer membrane permeabilization and the release of apoptosis-inducing factor-1 (AIF-1) [133]. AIF binds to macrophage migration inhibitory factor (MIF) and forms a complex that reaches the nucleus and causes DNA fragmentation and chromatin condensation, leading to cell death [134].

During sepsis, both the ischemic injury and the accumulation of ROS can trigger DNA fragmentation and subsequent parthanatos [135, 136]. Increased serum AIF levels of septic patients was associated with a 3-fold increase in mortality, suggesting the role of this mechanism in sepsis and the potential for its inhibition [137]. Therefore, PARP-1 inhibitors could pose a potential therapeutical approach in sepsis [138]. Olaparib, a PARP-1 inhibitor, shows potential clinical benefit in improving sepsis-outcome, by modulating the inflammatory cytokines production and maintaining host-defence mechanisms [139]. When delivered to the intestine of septic mice it reduced the formation of pro-inflammatory cytokines, bacterial translocation and improved survival rates [140]. By inhibiting PARP-1 and reducing mitochondrial damage, it shows anti-ferroptosis properties and improves cardiac function and survival in sepsis [141]. Olaparib also reduces inflammation and bacterial translocation via inhibition of extracellular-signal-regulated kinase (ERK)-pathway, a mechanism independent from parthanatos [142, 143]. Other parthanatos inhibitors are evaluated in clinical trials for several forms of cancer, but so far their effect in sepsis was not studied [144–147].

### MPT-Briven Necrosis, NETosis and Lysosome-Dependent Cell Death

#### MPT-Driven Necrosis

Mitochondrial permeability transition (MPT)-driven necrosis is a form of regulated cell death caused by perturbations of the intracellular environment. Increased ROS and Ca^2+^ inside the cell leads to the formation and opening of permeability transition pore complex (PTPC), leading to ATP depletion, osmotic swelling and plasma membrane rupture [148]. Although the exact mechanism of PTPC formation is not known, a key molecule involved in MTP-driven necrosis is peptidylprolyl isomerase F or cyclophilin D (CYPD) [5, 149]. The loss of mitochondrial membrane integrity leads to halted ATP synthesis causing an energy crisis that prevents apoptotic execution and shifts towards necrotic death [150]. This releases DAMPs, such as mitochondrial DNA and cytochrome c to amplify the inflammatory responses.

During sepsis the energy crisis caused by lack of perfusion, toxic metabolites and ROS leads to mitochondrial dysfunction that causes cytopathic hypoxia, cell death and ultimately organ damage [151, 152]. In a sepsis model of LPS-induced lung injury on CYPD (-/-) mice, the animals showed improved survival, reduced cytokine levels and inflammatory markers and reduced lung injury when compared to CYPD (+/+) mice [153]. Similarly, administration in CLP-induced septic mice of cyclosporin A, a PTPC and subsequently MLP-driven necrosis inhibitor, reduced cardiac dysfunction, improved lung histology scores, improved mitochondrial function and increased survival in septic mice [154, 155]. Overall, these studies point the potential of CYPD inhibition in sepsis, via reducing MPT-driven necrosis and mitochondrial dysfunction [156].

#### NETotic Cell Death

Neutrophils are the key players in the host response to infection by reaching the infection site and killing pathogens via various mechanisms, including the formation of neutrophil extracellular traps (NETs). NETs release plays an important part in both pathogen clearance and propagation of the inflammatory response by releasing DAMPs. Their activation is also involved in sepsis-associated thrombosis and coagulopathy [157]. NETotic cell death or NETosis represent a NADPH oxidase-mediated ROS-induced cell death present in hematopoietic cells that can produce extracellular traps (neutrophils, eosinophils and basophils) [5, 158]. LPS can induce NET formation and NETosis via GSDMD, one of the key molecules of pyroptosis, linking these two forms of cell death [159]. It is unclear whether necroptosis is also directly related to NETosis. While some studies linked RIPK1/3 and MLKL signaling to NET formation, others have shown that these can happen independently of RIPK3 or MLKL [160, 161]. NETosis inhibition reduced fibrin deposition in lung tissues, reduced key organs damage (lung, liver, kidney and heart) and improved overall survival in septic mice [162].

#### Lysosome-Dependent Cell Death

Lysosome-dependent cell death (LDCD) is a regulated form of cell death characterized by lysosomal membrane permeabilization (LMP) and the release of lysosomal hydrolases, particularly cathepsins, into the cytoplasm. LDCD is initiated by ROS, lipid peroxidation and specific pro-apoptotic proteins (e.g., Bax, Bid), that induce lysosomal destabilization and membrane rupture. The released lysosomal hydrolases (cathepsin B, D and L) degrade the cytoplasmatic and nuclear substrates, leading to cell death via MPT-driven necrosis, necroptosis and apoptosis [163, 164]. Also, because lysosomes contain large amounts of iron, their breakdown can lead to increased intracellular iron and subsequent ferroptosis [165, 166].

### Alkaliptosis, Disulfidptosis, Oxeiptosis, Cuproptosis and Paraptosis

Additional forms of regulated cell death (RCD), such as alkaliptosis, disulfidptosis, paraptosis, oxeiptosis and cuproptosis have also been identified, but for several of them their involvement in sepsis is not yet clear.

#### Alkaliptosis

Alkaliptosis is a form of RCD characterised by an increase in intracellular pH that can happen via the NF-kB-CA9 or the ATP6V0D1-STAT3 pathways. Carbonic anhydrase 9 (CA9) catalyses the reversible hydration of carbon dioxide into bicarbonate and a proton and is involved in maintaining an acidic environment in cancer cells [167]. NF-kB activation downregulates CA9, which leads to the formation of an alkaline intracellular environment and alkaliptosis [168]. ATP6V0D1 is a member of the vacuolar ATPase family that by binding to lysosomal STAT3 regulates the acidification of intracellular organelles. It does this by decreasing intralysosomal pH and thus increasing the intracytosolic one [169]. The main studied inducer of alkaliptosis is JTC-801, a high affinity, selective opioid acting on the NOP receptor [170]. It is used in animal pain-studies, but, because of its ability to induce alkaliptosis it is now tested in cancer models [168]. This form of cell death has been largely studied in cancer, where it could lead to tumour suppression and its relation to sepsis has not yet been validated. However, because of the regulatory role of NF-kB and the fact that sepsis patients, while usually presenting with extracellular acidosis, could also develop intracellular alkalosis in later phases of the disease, alkaliptosis could also be involved in septic injury and its inhibition could be a potential therapeutical target [171].

#### Disulfidptosis

Disulfidptosis is a form of RCD present in cells with high cystine accumulation. This happens under conditions of glucose-deprivation depletion of NADPH in cells with high activity of SLC7A11. Both mechanisms increase cystine levels by either reducing the amount transformed into cysteine or by increasing cellular uptake. Increased levels cystine and disulfide molecules promotes the formation of abnormal actin proteins in the cytoskeleton that ultimately leads to actin network collapse and cell death by disulfidptosis [172]. Because during sepsis both the cellular glucose-uptake and the antioxidant mechanisms are altered disulfidptosis could be a mechanism of organ injury [173]. Genetic studies have proposed disulfidptosis gene expression as markers of sepsis, septic lung injury and sepsis prognostic, however, their implication for patient management and as therapeutic targets remains to be validated [174–176].

#### Oxeiptosis

Oxeiptosis is a type of cell death that is triggered by the presence of excessive levels of ROS. While under physiological ROS levels the antioxidant mechanism KEAP1-Nrf2 induces the formation of antioxidant enzymes, under higher ROS levels KEAP1 suffers a conformational change [177, 178]. This leads to KEAP1 interaction to mitochondria-tethered phosphatase PGAM5 and the mitochondrial apoptosis inducing factor 1 (AIFM1), where KEAP1 is displaced from the mitochondria. PGAM5 further dephosphorylates AIFM1 that enters the cell nucleus and leads to cell death [179]. Although there is scarce data linking oxeiptosis to sepsis, it could represent an important RCD because of the increased ROS present during the early ischemia-reperfusion phase.

#### Cuproptosis

Cuproptosis is a copper-dependent form of regulated cell death in which excess mitochondrial Cu reduced by Ferredoxin 1 (FDX1) binds to lipoylated enzymes of the TCA cycle, causing their aggregation and destabilization of iron–sulfur (Fe–S) cluster proteins, which leads to proteotoxic stress and ultimately cell death [180]. Recent evidence suggests that cuproptosis contributes to organ injury in sepsis via dysregulated copper homeostasis, mitochondrial dysfunction and oxidative stress [181, 182]. In a septic acute kidney injury model reduced glutathione ameliorated tissue damage by lowering copper uptake and suppressing the cuproptosis pathway [183]. Current bioinformatic analyses suggests that cuproptosis-related molecules and gene expressions could be used as potential biomarkers in sepsis and that cuproptosis might be involved in sepsis-associated cardiomiopathy [184, 185].

#### Paraptosis

Paraptosis is characterised by the formation of large cytoplasmic vacuoles. These are formed by the swelling of the endoplasmic reticulum and mitochondria following Ca2 + and subsequently water influx. This ultimately causes the rupture of the outer membrane and cell death [186–188]. In a retinal ischemia/reperfusion injury model, retinal ganglion cells exhibited cytoplasmatic vacuolization at 6 h after the injury, with the main vacuoles being derived from the endoplasmic reticulum and/or mitochondria, a hallmark of paraptosis [189]. While thus it seems that paraptosis could be involved in ischemia/reperfusion organ injury, its role in sepsis has not yet been studied.

### Regulated Cell-Death Cross-Network

**Fig. 4:**
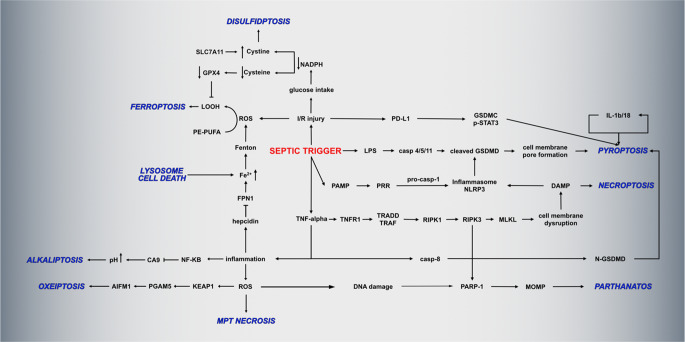
Integration of non-apoptotic regulated cell death mechanisms in sepsis. This is a schematic representation of the in-text presented processes: the septic trigger causes a cascade of reactions: release of LPS, PAMPs, inflammatory reactions and I/R injuries. These lead to reactions that culminate in several interconnected forms of regulated cell death, that lead to the propagation of inflammation and tissue injury. *Abbreviations*: AIFM1, Apoptosis-inducing factor 1, mitochondrial; CA9, Carbonic anhydrase 9; casp, caspase; DAMP, damage associated molecular pattern; FPN, ferroportin; GPX4, Glutathione peroxidase 4; GSDMC, gasdermin C; GSDMD, gasdermin D; IL, interleukin; I/R, ischemia/reperfusion; KEAP1, kelch like ECH associated protein 1; LOOH, lipid hydroperoxide; LPS, lipopolysaccharide; MLKL, Mixed Lineage Kinase Domain-Like protein; MOMP, Mitochondrial Outer Membrane Permeabilization; MPT, Mitochondrial Permeability Transition Pore; NADPH, Nicotinamide adenine dinucleotide phosphate; NF-kB, Nuclear factor kappa-light-chain-enhancer of activated B cells; NLRP, Nucleotide-binding oligomerization domain, Leucine rich Repeat and Pyrin domain containing; PAMP, pathogen-associated molecular pattern; PARP, Poly(ADP-ribose) polymerase; PD-L, Programmed Death-Ligand; PE-PUFA, phosphatidylethanolamine-polyunsaturated fatty acids; PGAM5, Phosphoglycerate mutase familymember 5; PRR, Pattern recognition receptors; RIPK, Receptor Interacting Protein Kinases; ROS, reactive oxygen species; SLC7A11, glutamate/cystine antiporter solute carrier family 7 member 11; TNF, tumoral necrosis factor; TNFR, tumoral necrosis factor receptor; TRAF, Tumor Necrosis Factor Receptor Associated Factors; TRADD, Tumor necrosis factor receptor type 1-associated DEATH domain protein

During sepsis the ischemia/reperfusion injury triggers a cascade of reactions, that culminate with the activation of several cell death mechanisms [190]. Many of these RCD mechanisms are interconnected and thus share certain pathways and molecules that are schematically represented in Fig. 4. For example PANoptosis refers to the interrelationship between pyroptosis, apoptosis and necroptosis [191]. PANoptosis plays a central role in sepsis by amplifying inflammation, impairing immune cell function, and driving multi-organ dysfunction. Recent studies highlight its involvement in the dysregulated immune response characteristic of sepsis [192, 193].

The septic trigger leads to a miriad of reactions, including ischemia/reperfusion injury and local inflammatory reactions. These lead to the formation of ROS that are involved in both producing lipid peroxides, that lead to ferroptosis, as well as to KEAP1-mediated PGAM5-AIFM1 oxeiptosis and MPT-driven necrosis. The antioxiadative system GSH-GPX4 that could clear the excessive ROS and prevent from ferroptosis depends on SCL7A11 to import into the cell cystine. But during conditions of decreased glucose intake, as it happens during sepsis, the depletion of NADPH leads to less formation of cysteine from cystine, hindering the GPX4 system, but also leading to increased cystine accumulation and thus disulfidptosis. Also, because in sepsis there is an increase in hepcidin activation, this leads to iron sequestration and increased ROS formation via the Fenton reaction. The free iron pool can also increase because of lysosome cell death. Also, increased copper can not only lead to cuproptosis, but also to ferroptosis. Copper chelators in sepsis have reduced the ferroptotic injury by reducing copper-related GPX4 degradation, but had no effects on other forms of cell death [194]. On the other hand, inflammatory mediators such as TNF-α and NF-kB lead via a series of reactions to pyroptosis, alkaliptosis, necroptosis and parthanatos. All of these RCD release a series of DAMPs and pro-inflammatory molecules that amplify the tissular damage, ROS release and inflammation.

Therefore, it is difficult to discuss one RCD without having in consideration the others with which it interconnects. Future research should try to discuss these mechanisms together as a RCD network of events in sepsis-related organ damage and should try to focus on complex treatment strategies that can target multiple pathways. Also, diagnostic studies should focus on the amount of activation of each cell death pathway during the evolution of the disease. It is possible that cell death pathways such as ferroptosis and pyroptosis to be activated in the early phases of the disease, while others such as alkaliptosis to be involved in late-sepsis damages.

## Current ICU Medication and its Effect on Regulated Cell Death Mechanisms (Table 2)

During the ICU stay and especially during sepsis, patients require several classes of medication, including fluids, antibiotics, hypnotics and analgesic medication. All these could exert an effect on the molecular mechanisms that underly the septic injury. In this section we will discuss the most-used ICU medication and their effects on RCD and since several of them were not studied on septic patients or septic murine models, we have also included studies that look at other pathologies (Table 2).

**Table 2:** ICU-drugs effects on regulated cell death mechanisms. Abbreviations: AKT, protein kinase B; CoQ10, coenzyme Q10; eNOS, endothelial nitric oxide synthase; Fe²⁺, ferrous iron; FSP1, ferroptosis suppressor protein 1; GPX4, glutathione peroxidase 4; GSDMD, gasdermin D; GSH, glutathione; GSH-Px, glutathione peroxidase; GSK3β, glycogen synthase kinase 3 beta; HIF-1α, Hypoxia-inducible factor 1-alpha; HMGB, high mobility group box; HO-1, Heme oxygenase-1; HSP90, heat shock protein 90; I/R, Ischemia/reperfusion; IL, Interleukin; KEAP1, Kelch-like ECH-associated protein 1; MLKL, mixed lineage kinase domain-like protein; MPT, mitochondrial transition pore; NLRP3, NOD-like receptor family Pyrin domain–containing 3; NO, nitric oxide; Nrf2, nuclear factor erythroid 2–related factor 2; PARP1, Poly(ADP-ribose) polymerase 1; PI3K, phosphoinositide 3-kinase; RIP, Receptor-interacting protein; ROS, reactive oxygen species; SIRT1, Sirtuin 1; SLC7A11, solute carrier family 7 member 11; STAT3, signal transducer and activator of transcription 3; TNF, tumor necrosis factor

Drug	Effect on Regulated Cell Death	Molecular Mechanism	Organ	References
Dexmedetomidine	Inhibits **ferroptosis**	Increases the binding of KEAP1 and HSP90 to GPX4, reducing GPX4 degradation; Promotes the Nrf2/SLC7A11/FSP1/CoQ10 pathway	Septic renal injury; Sepsis-Induced Vascular Leakage	[195–197]
	Inhibits **pyroptosis**	Activates the PI3K/AKT/GSK3β pathway; Inhibits caspase-1, IL-1β and IL-18; Reduced NLRP3, cleaved-caspase-1, GSDMD, IL-1β, and IL-18	Early Brain Injury After Subarachnoid Hemorrhage; Septic brain injury; Myocardial I/R injury	[198–200]
	Inhibits **parthanatos**	Suppresses ROS/NLRP3 pathway; Inhibits PARP1	Cardiomyocites; Human neuroblastoma cells	[201, 202]
	Promotes **necroptosis**	Upregulates PARP1	non-small cell lung cancer	[203]
	Inhibits **necroptosis**	Decreases RIP1, RIP3, MLKL, HMGB1, and ROS levels;	Septic liver injury; Cardiomyocytes	[204, 205]
Propofol	Inhibits **ferroptosis**	Upregulates Nrf2; Increases GPX4 levels; Modulates eNOS/NO signaling pathway	Sepsis-Induced Brain Injury; Myocardial I/R injury; Traumatic brain injury	[206–208]
	Promotes **ferroptosis**	Downregulates STAT3	Colorectal cancer cells	[209]
	Inhibits **pyroptosis**	Upregulates mTOR pathway; Downregulates Caspase-1 and GSDMD; Inhibited NLRP3 activation	Acute lung injury; Traumatic brain injury	[210–212]
	Promotes **pyroptosis**	Increases NLRP3/caspase-1	Developing rat brain neuroinflammation; Murine macrophages	[213–215]
	Inhibits **necroptosis**	Upregulates SIRT1	Renal I/R injury	[216]
	Inhibits **parthanatos**	Decreased ROS overproduction, calcium release from ER, and mitochondrial depolarization	Cerebral murine cell culture	[217]
	Inhibites **MPT-driven necrosis**	Inhibited MPT pore opening	Myocardial I/R injury	[218]
Remimazolam	Inhibits **pyroptosis**	Inhibited NLRP3;	Myocardial I/R injury; Cerebral I/R injury	[219, 220]
Fentanyl	Promotes **pyroptosis**	Activated NLRP3 inflammasome, caspase-1 and GSDMD	Repeated administration on Dorsal Raphe Nucleus neurons	[221]
Remifentanil	Promote **pyroptosis**	Increased Ca^2+^, ROS and NLRP3 via calcium-sensing receptor activation	Myocardial I/R injury	[222]
Sufentanil	Inhibits **ferroptosis**	Increased Nrf2 via the Akt/GSK-3β pathway; Downregulates the expression levels of HIF-1α	Cerebral I/R injury; Hepatic I/R injury	[223, 224]
Morphine	Inhibits **necroptosis**	activating PKCε-ERK1/2 pathway and	Myocardial I/R injury	[225]
	Inhibits **MPT-driven necrosis**	inhibits mitochondrial permeability transition pore opening	Myocardial I/R injury	[226]
Oxycodone	Inhibits **pyroptosis**	Increases Nrf2	LPS-induced myocardial injury	[227]
Ketamine	Inhibits **ferroptosis**	Increases GSH-Px and GSH and decreases IL-6, TNF-α, MDA, Fe2+; Increased the expression of HIF-1α/HO-1 pathway	Cardiomyocite oxygen-glucose deprivation/reoxygenation; LPS induced acute lung injury	[228, 229]
	Promotes **ferroptosis**	Inhibited the expression of GPX4	Breast cancer cells	[230]
	Promotes **pyroptosis**	Activates NLRP3/Caspase-1 pathway	Renal cells in developing rats; Hippocampus of developing rats	[231–233]
Acetaminophen	Inhibits **ferroptosis**	Upregulates GPX4 and FSP1	Septic-brain injury;	[234, 235]
Epinephrine	Inhibits **ferroptosis**	Decreased iron and ferritin levels, diminished cytokine storm	SARS-CoV-2 case	[236]
Vasopressin	Promotes **ferroptosis**	Increases ACSL4, Fe 2 + concentration, and MDA level	Human cardiomyocytes	[237]

### Dexmedetomidine

Dexmedetomidine (DEX) is a selective α2-adrenoceptor agonist with sedative, analgesic and opioid-sparing effects. It is suitable for use in the intensive care for sedation of patients with sepsis that require mechanical ventilation [238, 239]. Besides these properties, DEX reduces inflammation and ferroptosis [240, 241]. In sepsis models, DEX seems to exert the protective effects via its α2-adrenoceptor agonist effect. This was demonstrated in a model of septic myocardial injury where DEX alone reduced the inflammatory and lipid peroxidation damage caused by ferroptosis activation and the administration of an α2-adrenoceptor antagonist reduced the beneficial effects of DEX [242]. Also, by reducing pyroptosis, DEX reduced myocardial ischemia/reperfusion injuries in septic rats [243]. In ischemia/reperfusion myocardial injury, DEX inhibited pyroptosis and increased cell viability [200]. Similar results were seen in septic encephalopathy models, where DEX had protective effects via central α2-adrenoceptor activation and by inhibiting pyroptosis [199, 244]. Also, by inhibiting pyroptosis in hypoxic-ischemic and neuroinflammatory injuries DEX reduced neuronal apoptosis and alleviated cerebral damage [198, 245]. Protective effects were also observed on sepsis-induced vascular leakage via a ferroptosis-inhibitory mechanism by upregulation of Nrf2 [196, 197]. In renal injury DEX had a protective effect by reducing GPX4 degradation and ferroptosis [195].

In non-small cell lung cancer cells DEX increased PARP1 and promoted necroptosis, leading to cell death [203]. However, in a myocardial ischemia/reperfusion model, DEX preconditioning inhibited necroptosis and inflammation, providing a protective effect [205].

DEX also inhibits parthanatos by regulating PARP1 expression [202]. In a chronic heart failure model DEX inhibited ROS formation, NLRP3 inflammasome and PARP1 activation and reduced parthanatos and cell death [201].

### Propofol

Propofol is one of the most used sedation and hypnotic agents in the ICU. In an LPS-SAE model it showed beneficial anti-ferroptosis properties by activating the expression of Nrf2/HO-1 axis, reduced ROS levels and lipid peroxidation and increased GSH and GPX4 antioxidant systems [206].

However, it can induce pyroptosis of primary hippocampal neurons via NLRP3 expression and pyroptosis. These effects were counteracted by prior administration of DEX to the cells [214]. Moreover, high-dose propofol can induce pyroptosis via caspase-1 cleavage and NLRP3 activation [215]. In developing rats, the neuroinflammation induced by propofol was reduced by inhibiting NLRP3 activation, linking the propofol-induced cognitive impairment to pyroptosis activation [213]. Interestingly, in cigarette smoke-induced lung injury, propofol reduced the inflammatory response and cell death by decreasing NLRP3 expression and pyroptosis [246]. Similarly, in renal ischemia/reperfusion-induced acute lung injury propofol reduced inflammation, NLRP3 and caspase-1 [212]. The same study group showed that in this model propofol also inhibited necroptosis [216]. However, in a LPS-model propofol did not reduce intestinal enterocytes damage or improve survival by inhibiting pyroptosis [247].

In ischemia/reperfusion models propofol could reduce ROS generation and aberrant calcium release, while also reducing mitochondrial damage and parthanatos [217]. By reducing ROS, it could also contribute to lower MPT-driven necrosis. In a heart-ischemia model propofol reduced ROS and MPT pore formation, providing beneficial effects [218]. Also, it diminished the mitochondria swelling in cerebral ischemia/reperfusion injuries in rat [248].

Additionally, another sedative drug, remimazolam used for procedural sedation presented an anti-pyroptotic effect by inhibiting NLRP3 in cerebral ischemia/reperfusion injury in rats [220].

### Fentanyl, Remifentanil, Sufentanil

Besides sedation, analgesia is a crucial component in ICU patients’ sedation. Among the available strategies, opioids are the most useful thanks to their high efficacy [249]. However, several studies linked opioids to immunodepression, increased risk of infection or even reduced ability of the innate immune system to fight off infections [250]. In the dorsal Raphe nucleus, fentanyl and morphine caused an increase in GSDMD levels, NLRP3 activation and pyroptosis [221]. However, delta-opioid receptor activation could potentially reduce TNF-α release and reduce necroptosis and inflammation [251]. In a cerebral ischemia/reperfusion injury model sufentanil decreased neuronal ROS formation, inflammation and ferroptosis via a Nrf2-mechanism and protected the cerebral tissue [223]. By inhibiting ferroptosis, sufentanil also decreased hepatic ischemia/reperfusion injury in mice and downregulated the expression levels of HIF-1α [224]. Opioid receptors agonists could also be beneficial in reducing cell death in myocardial ischemic injuries [252]. Chen et al. showed that in myocardial ischemia/reperfusion injury, morphine postconditioning reduced infarct size and improved cardiac recovery via the inhibition of MPT-driven cell death [225, 226]. Additionally, oxycodone in a LPS-sepsis model reduced myocardial ROS, inflammation and pyroptosis [227]. High-doses of remifentanil can however increase myocardial ischemia/reperfusion injury by increasing ROS and NLRP3 activation and pyroptosis [222].

### Ketamine

Ketamine is a NMDA receptor antagonist with uses in sepsis as both sedation agent and analgesic adjuvant [253]. Regarding cell death mechanisms, ketamine induces ferroptosis in cancer cells models and increases ROS, pyroptosis and ferroptosis in the cerebral tissue of newborn rats [232, 253, 254]. However, in sepsis models it could be protective against lung injury and inflammation [255]. By modulating the HIF-1α/HO-1 pathway, esketamine, the S(+) isomer of ketamine, reduces ferroptosis in LPS-induced lung injury and reduces ROS and inflammation in cardiac injury caused by tourniquets [228, 229]. Low dose esketamine can also reduce the levels of IL-1β, GSDMD and NLRP3 and reduce pyroptosis in LPS-treated microglial cells [256].

### Acetaminophen

As a commonly used antipyretic agent, acetaminophen (APAP) is frequently used in septic patients with high fever [257]. APAP also has other benefits in sepsis, including reducing lipid peroxidation and ferroptosis via increased GPX4 expression [235]. These results show APAP as a possible therapeutic adjuvant against sepsis encephalopathy by inhibiting ferroptosis [234]. A recent clinical trial that tested APAP as a possible preventive treatment for organ dysfunction of ICU patients yielded unsignificant results [258].

### Vasoactive Drugs

In a case report of SARS-CoV-2 infection, epinephrine reduced ROS and the cytokine storm, partially by inhibiting ferroptosis [236]. However, more studies are needed to confirm this mechanism.

In a heart failure model, vasopressin increased ferroptosis, it reduced GPX4 levels and increased ACSL4, MDA and iron levels [237].

### Vitamin D, E and Selenium

Vitamin D supplementation has been proposed as a potent inhibitor of inflammation through its active metabolism and via the activation of vitamin D receptor on immune cells [259]. This effect also involves ferroptosis inhibition and it has been confirmed in several in vivo experimental sepsis studies [260]. In the liver of septic mice, calcitriol administration led to lowered inflammation and reduced ferroptosis markers and genes expression [261]. Also, vitamin D receptor upregulation decreased the sepsis-associated intestinal injury in a rat septic model [262]. Up to date, the clinical trials and systematic reviews involving critically ill septic patients that received vitamin D supplementation showed no improvement of their disease progression [263, 264].

Vitamin E is a natural antioxidant that is reduced in critically ill patients [265]. Its supplementation could be beneficial by reducing lipid peroxidation and ferroptosis [266, 267]. Experimental studies showed that in sepsis, vitamin E administration could reduce the effects of septic lung-damage [268]. A meta-analysis showed that vitamin E administration could reduce ICU lengths of stay, but had no significant effect on mortality, duration of mechanical ventilation or frequency of adverse events [269].

Selenium augments the formation of selenoproteases, of which GPX4 that is involved in ferroptosis could pose a clinical benefit in reducing the cellular oxidative stress and organ damage [270, 271]. Although experimental studies showed a clear benefit in septic animal models, systematic reviews of selenium supplementation showed no benefit in regards to incidence of renal failure or duration of mechanical ventilation, but there was a reduced vasopressor therapy duration and reduced ICU hospitalization [268, 272, 273]. Although the current literature data is sparse, future studies involving selenium administration could prove beneficial, especially in subgroups of patients where there is a high inflammatory response.

## Conclusions and Future Perspectives

Immunogenic cell death mechanisms are an important piece in the puzzle of sepsis, being involved in the initial inflammatory response and associated organ injury, but also in bacterial and viral infected cell clearance and in the subsequent immune paralysis phase. Their inhibition or stimulation by current ICU medication does not seem to pose real importance in current practice, but it should be studied more to find better alternatives with no/lower side effects.

When considering the current literature, there is a trend that inhibiting cell death mechanisms, whether ferroptosis, pyroptosis, necroptosis or other immunogenic cell death mechanism leads to an overall reduced inflammatory response, reduced organ damage and improved survival. Most of these studies, however, use acute models of sepsis, meaning that they do not analyse the long-term immunosuppression that could arise from following such treatment. Also, they do not consider which animals were or were not susceptible to these therapies, i.e. which one of them had increased inflammatory markers at the time of treatment administration. Another weakness of our current models is that many of them lack the antibiotic and fluid resuscitation treatment that could reduce the initial ischemic injury and render cell-death inhibitors insignificant.

Since RCD are tightly connected to sepsis-associated organ damage, targeting these processes or using them as biomarkers could be useful research avenues. RCD markers should also be studied in the evolution of the disease and classified as alterations in early- and late-sepsis, in order to better tailor RCD inhibitors or inducers. To date, there are no ongoing clinical trials of specific RCD inhibitors in sepsis.

Future research should first improve the animal models, using more complex ones that better replicate complex human-management, including at least fluid resuscitation and prophylactic antibiotic treatment. It should also focus on stratifying the results based on an initial assessment of the inflammatory status using current biomarkers (IL-6, TNF-α) and cell-death markers. Finally, the inhibition of cell death pathways should be assessed not only at the systemic level in sepsis, but also within specific organs and key cell types, to determine where such interventions may be beneficial and where they might prove harmful.

Non-apoptotic regulated cell death mechanisms are involved in septic and ischemia/reperfusion injuries leading to the propagation of inflammation, reactive oxygen species and organ damage. Researching these pathways could be beneficial in developing adjuvant targeted therapies for improving sepsis outcomes or as new biomarkers in evaluating sepsis progression and severity.

## Data Availability

No datasets were generated or analysed during the current study.
